# Low Seroprevalence of WNV in Namibian Dogs Suggests a Limited Effectiveness as Sentinels for Infection Monitoring

**DOI:** 10.3390/tropicalmed8040203

**Published:** 2023-03-29

**Authors:** Umberto Molini, Giovanni Franzo, Barbara Bonfini, Lourens de Villiers, Mari de Villiers, Siegfried Khaiseb, Federica Monaco, Giovanni Savini, Nicola D’Alterio

**Affiliations:** 1 School of Veterinary Medicine, Faculty of Health Sciences and Veterinary Medicine, University of Namibia, Neudamm Campus, Windhoek 13301, Namibia; 2 Central Veterinary Laboratory (CVL), 24 Goethe Street, Windhoek 18137, Namibia; 3 Department of Animal Medicine, Production and Health, University of Padova, Legnaro, viale dell’Università 16, 35020 Padova, Italy; 4 Istituto Zooprofilattico Sperimentale dell’Abruzzo e del Molise, 64100 Teramo, Italy

**Keywords:** West Nile virus, dogs, Namibia, prevalence, serology

## Abstract

West Nile virus (WNV) is an important zoonotic Flavivirus responsible for mild fever to severe neurological disease in humans and horses. Despite the occurrence of major previous outbreaks in Namibia and the likelihood of the current endemicity of the virus, only limited investigations and monitoring activities of WNV have been performed in the country. The use of animal sentinels is a valuable approach toward investigating the infection presence in an area and to predict the potential occurrence of human outbreaks. Serological investigations in dogs hold several advantages, considering their infection susceptibility, the ease of sample handling, and the evaluation of risk factors of pet owners that share the same habit with their pets. To evaluate the usefulness of such a sero-epidemiological investigation in Namibia, a broad serosurvey was performed in 2022 that included 426 archived domestic dog samples from eight Namibian regions. Although the ELISA prevalence, indicative of Flavivirus infection, was relatively high (16.43%; 95 CI: 13.10–20.39%), the virus neutralization test confirmed only a minority of cases, highlighting a prevalence of 2.82% (95 CI: 1.47–4.90%), significantly lower than in Namibian donkeys and reports from other countries. Variables that could explain the recorded differences remain to be explored, including animal exposure, variable vector presence, distribution, and feeding preferences. The study results suggest the limited usefulness of dogs as sentinels for WNV monitoring in Namibia.

## 1. Introduction

Initially described in 1937 in Uganda, West Nile virus (WNV) is currently recognized as a relevant human pathogen with severe consequences for human health in different parts of the world. WNV, belonging to the family *Flaviviridae*, genus *Flavivirus*, is a mosquito-borne virus maintained in nature through an enzootic cycle involving ornithophilic mosquitos and birds. Other susceptible species, that represent incidental hosts not involved in the infection cycle, include horses and humans that may as a result be afflicted with severe clinical consequences. An estimated 20% of infected people show clinical signs and approximately 1% develop neurologic illness or other syndromes. Nevertheless, the consequences of infection may be severe and impact on human health, welfare, and productivity. About 10% of the neuroinvasive cases are reportedly fatal and recovery from severe illness might take several weeks to months, with more than 50% of survivors presenting physical and cognitive sequelae up to 2 years later [1,2].

Moreover, especially in countries such as South Africa where horse farming is an important source of income, WNV can have a remarkably detrimental effect considering that ∼10% of infected horses develop clinical signs, with mortality rates as high as 50%. Around 10–20% of horses that recover from neurological disease retain residual neurologic deficits [3].

For these reasons, an effective surveillance system should be mandatory in endemic regions. Unfortunately, surveillance activities are limited in low-income countries, where diagnostic activity is limited and the infection and disease rates are underreported both in human and animal cases. Several domestic and wild animals are susceptible to the infection [4,5,6] and could be used as sentinels for signaling regions at risk of virus transmission to humans, especially in resource-poor settings where facilities for human disease detection might be lacking.

To be effective, sentinels must have several prerequisites besides susceptibility:

(a) Develop a strong and long-lasting antibody response that can be easily detected;
(b) Have low levels of viremia, thereby not participating in the propagation of the infection and minimizing the risk to operators;
(c) Be distributed in the region(s) of interest and be able to be easily sampled;
(d) Ideally, share the same environment and risk/exposure factors as human beings to maximize their representativeness and predictive power.

Domestic pet dogs seem to satisfy the requirements to be effectively evaluated as sentinels in different parts of the world, demonstrating a high level of seroprevalence and good predictive performances on the emergence of human cases [7,8,9,10,11]. However, the structure of the dog population and pet–owner interactions could affect the representativeness of domestic dogs as disease sentinels for WNV. Similarly, the feeding preferences of mosquitoes on dogs could be influenced by the prevalent mosquito species, environmental features, and the presence of other hosts in the area/region investigated. Therefore, a careful assessment should be performed to evaluate the applicability of dogs as sentinels for WNV circulation and early detection in particular geographical regions. In this study, an extensive serological study was performed in several Namibian regions in domestic dogs to evaluate the real seroprevalence and compare it with other equine species previously considered as potential sentinels.

## 2. Materials and Methods

### 2.1. Sample Collection

Archived serum samples, collected between January and September 2022 from domestic dogs during veterinary consultation activities at the Veterinary Academic Hospital (VAH) of the University of Namibia, were evaluated. The canine samples originated from 8 different regions of Namibia, namely Erongo, Hardap, Karas, Kavango West, Khomas, Kunene, Omaheke, and Otjozondjupa (Table 1). The VAH provides veterinary services to the general public, but also provides mobile veterinary services to low-income, rural-living communities in remote locations that cannot afford private veterinary services. Therefore, a representative sample of the Namibian canine population could be achieved. The sample size was calculated to estimate the infection prevalence with a precision of 5% and a confidence of 95%, assuming an estimated prevalence level of 50% in the absence of any large-scale serological studies on WNV disease prevalence in the country. The minimum sample size was calculated as being 380 dogs; however, a higher number of previously archived samples were available. Metadata on location, gender, age (<1, 1–7, and >7 years), and breed (pure or crossbred) were included for each animal.

Serum was separated via centrifugation at 8000 rpm for 10 min and stored at −20 °C until analysis. Serum samples were screened for flavivirus antibodies using a commercial competitive ELISA (cELISA) (ID Screen^®^ West Nile Competition Multi-species, IDvet, Grabels, France) in accordance with the manufacturer’s instructions. The cELISA targeted the antibodies against one epitope of the Pr-E protein of Flavivirus. Positive samples were confirmed using a virus neutralization test (VNT) in microtiter plates for the detection of specific neutralizing antibodies against WNV, in accordance with the World Animal Health (OIE) Manual of Diagnostic Tests 2021 [12,13]. The serum samples were diluted, starting from dilution titer 1:5 to 1:640, and an equal volume of 100 µL TCID50 (tissue culture infectious dose) reference WNV field strain was added to each dilution. After 1 h at 37 °C and 5% CO_2_ in a humidified incubator, 100 µL of 10^5^ Vero cells was added to each well. The plates were incubated at 37 °C for 5 d. Starting from the third day after incubation, the plates were checked for cytopathic effect (CPE). The positive threshold was set at the 1:10 dilution. The sample was considered to be positive when it showed more than 90% CPE neutralization at the lowest dilution (1:10). ELISA and VNT analyses were performed at the OIE Reference Laboratory for West Nile Disease, Istituto Zooprofilattico Sperimentale “G. Caporale”, Teramo, Italy.

### 2.2. Statistical Analyses

The association and the relative odds ratio between WNV serology results and animal gender, breed, age, and collection test were calculated by fitting logistic regressions using the ELISA and VNT results as the outcome variables. Seroprevalence and relative confidence intervals were calculated using the EpiR v2.0.56 package [14]. The association between rainfall in the considered regions and WNV seroprevalence was also assessed via linear regression. All of the analyses were performed in R v4.2.2. The statistical significance level was set to *p*-value < 0.05.

## 3. Results

A total of 426 archived dog samples were included in the study: 190 female and 236 male; 394 crossbreeds and 32 purebreds; and an age distribution of 106 younger than one year, 298 between the age of 1–7 years, and 22 older than 7 years.

A total of 70/426 (16.43%; 95 CI: 13.10–20.39%) analyzed dog samples tested positive via ELISA, and the presence of WNV neutralizing antibodies was confirmed via VNT in 12 animals (2.82%; 95 CI: 1.47–4.90%) (Table 1). Neutralizing titers of 1:10, 1:20, and >1:20 were found in four, five, and three animals, respectively. The circulation of WNV was detected in six out of eight regions under investigation with a prevalence between 1.56% and 9.3% (Table 1).

A significant association was detected between ELISA seropositivity and dog age, with animals between 1 and 7 years and older than 7 years having an odds ratio for positivity of 7.83 (95 CI: 2.81–32.65; *p*-value < 0.001) and 22.00 (95 CI: 5.97–106.91; *p*-value < 0.001) compared to younger dogs. A significant effect related to the region of sample origin was also observed, with higher prevalence in the Hardap (odds ratio = 4.26; 95 CI: 1.55–13.79; *p*-value = 0.007) and Kavango West (odds ratio = 5.11; 95 CI: 1.76–17.17; *p*-value = 0.004) regions compared to Khomas.

No significant association was found between WNV VNT results and any of the considered signalment variables (Table 2). A positive correlation was observed between average rainfall in the considered region and the ELISA (b = 0.019; R^2^ = 0.261; *p*-value = 0.196) and VNT (b = 0.014; R^2^ = 0.576; *p*-value = 0.029) seroprevalence.

## 4. Discussion

WNV disease prevalence is largely unknown in Namibia, and Africa in general, where facilities with the capacity and resources to conduct the diagnosis of Arbovirus infections are lacking [15]. The few studies that have been performed in Namibia for Flavivirus detection revealed seroprevalence of between 8 and 30.2% [16,17,18]. Scattered information from other sub-Saharan countries revealed highly variable seroprevalence values, ranging from less than 10% to more than 60%, and a remarkable diversity ascribed to the specific countries and ecosystems investigated [15,19,20]. While WNV is clearly endemic in Africa, necessitating an urgent need for measures that can survey disease prevalence to protect humans from such zoonotic pathogens, the adoption of appropriate measures (reviewed in [15]) is rarely applied. The monitoring of viral disease presence and spread is of fundamental importance to define and prioritize prevention and control measures for zoonotic diseases.

Horses and donkeys previously evaluated showed high WNV seroprevalence and could potentially act as effective sentinel species [3,21]. However, the distribution of equid sentinels is not necessarily representative of human exposure to disease, due to a more limited human–animal interface with equines when compared to domestic pets. Active sampling in equine sentinel animals may pose additional practical challenges for representative sample collection where resources are limited. Similar concerns can be raised in consideration of wild birds or mosquitos as sentinels.

On the contrary, domestic pet dogs share the same habitat and risk factors as their owners and routine veterinary medical examination could allow for easy and inexpensive sample collection as part of surveillance measures. Accordingly, dogs have been utilized as sentinels for zoonotic disease surveillance in many other countries. After the WNV epidemic of New York in 1999, dogs in Queens had a seroprevalence four times higher than human beings [7]. Moreover, dogs tested at an animal control facility in Huston revealed WNV-positive dogs 6 weeks prior to the first reported human cases, and the highest peak in human cases coincided with the peak of seropositivity in the dogs (~40%) [11]. Comparable evidence has been obtained in several other countries where sentinel animals were utilized successfully [8,10,11,16,22,23].

The current study results are in contradiction with the effective use of sentinel dogs for WNV disease monitoring, as reported in previous investigations. Although the ELISA-positive dog results were overall in line with other studies’ reported findings, only a minority of the ELISA-positive dogs were confirmed positive in this investigation through VNT by the OIE reference laboratory. While VNT is considered to be less sensitive, it must be stressed that the ELISA test used was not WNV-specific and cross-reactivity with other Flaviviruses was to be expected. Therefore, discrepant results could relate to possible cross-reactivity from dogs’ previous exposure to other related viruses, whose characterization and regional distribution may deserve further investigation.

VNT-based studies in dogs from other countries reported higher seroprevalence [7,8,10,11,23]; therefore, a truly lower WNV infection in Namibian dogs can be advocated. The possible reasons for the reported result differences in this investigation may prove difficult to clarify. A lower circulation of WNV in Namibia might occur, as supported by the relatively low prevalence reported in humans [16,18]. Environmental conditions as well as the species and feeding preferences of vectors can vary depending on the specific location [5,15,20]. Most of the Namibian samples originated from dry, desertic areas where a mosquito presence was limited. A positive correlation was observed between average rainfall in the considered regions and the ELISA and VNT seroprevalence (Figure S1). Accordingly, available matching data from donkey sentinels showed a relatively lower seroprevalence via VNT, while higher, albeit not statistically significant, values were reported for both species sentinels in areas such as the Kavango region [21].

Nevertheless, with account taken of the regions from where the samples originated, the seroprevalence was constantly lower in dogs from this study compared to equid sentinels in previous studies. A preferential feeding activity of the vectors on equids rather than dogs could be speculated. Unfortunately, data are limited on the occurrence and characterization of competent vector species in Namibia. The presence of *Aedes aegypti* and *Culex pipiens* has been described at six surveillance sites in Windhoek, of which only *C. pipiens* is an efficient WNV vector [2,5,18]. However, a remarkable variability in population composition was observed amongst sampling sites and times. Therefore, no reliable information could be derived for the regions considered in the present study based on previous studies, since other vectors with different biology might be present and relevant [18]. If the mosquito vector species present in the regions of interest prefer to feed on humans or equines, dogs may not be appropriate sentinels for such areas. Variability in vector exposure might also carry weight in areas where pet dogs reside mostly indoors or live in peri-urban areas where contact with mosquitos is less frequent.

## 5. Conclusions

The study results highlight the low prevalence of WNV in the Namibian dog population and imply a limited utility of domestic pet dogs, compared to equids, as early detection sentinels for WNV in the human population within the considered settings. Additional studies should be performed to evaluate the reservoir and vector presence as well as the features and distribution in Namibia to further clarify the possible causes behind the remarkable difference in this study’s results when compared to similar investigations performed in other non-African countries.

## Figures and Tables

**Table 1:** Seroprevalence of WNV in dogs according to Namibian regions.

Region	N. Dogs	cELISA	VNT WNV
Omaheke	64	6/64 (9.37%; 95 CI: 3.51–19.29%)	1/64 (1.56%; 95 CI: 0.039–8.40%)
Erongo	42	0/42 (0%; 95 CI: 0–8.41%)	0/42 (0%; 95 CI: 0–8.41%)
Khomas	51	5/51 (9.8%; 95 CI: 3.26–21.41%)	2/51 (3.92%; 95 CI: 0.47–13.45%)
Kunene	62	10/62 (16.13%; 95 CI: 8.01–27.66%)	2/62 (3.23%; 95 CI: 0.39–11.17%)
Otjozondjupa	64	9/64 (14.06%; 95 CI: 6.63–25.02%)	1/64 (1.56%; 95 CI: 0.04–8.40%)
Kavango west	43	15/43 (34.88%; 95 CI: 21.01–50.93%)	4/43 (9.3%; 95 CI: 2.59–22.13%)
Karas	40	6/40 (15%; 95 CI: 5.71–29.83%)	0/40 (0%; 95 CI: 0–8.81%)
Hardap	60	19/60 (31.67%; 95 CI: 20.25–44.95%)	2/60 (3.33%; 95 CI: 0.41–11.53%)
Total	426	70/426 (16.43%; 95 CI: 13.10–20.39%)	12/426 (2.82%; 95 CI: 1.47–4.90%)

cELISA = competitive enzyme-linked immunosorbent assay; WNV = West Nile virus; VNT = virus neutralization test.

**Table 2:** Odds ratio, 95 CL, and *p*-value for the different risk factors calculated for the ELISA and VN tests.

Test	Variable (Reference Level)	Target Level	Odds Ratio (CI 95%)	*p*-Value
ELISA	Sex (female)	Male	1.34 (0.80–2.31)	0.267
	Age (age < 1 year)	Age 1–7 years	7.83 (2.809–32.65)	<0.001
		Age > 7 years	22.00 (5.97–106.91)	<0.001
	Breed (crossbreed)	Purebred	1.46 (0.56–3.36)	0.398
VN	Sex (female)	Male	1.06 (0.33–3.66)	0.921
	Age (age < 1 year)	Age 1–7 years	2.17 (0.09–23.64)	0.572
		Age > 7 years	1.56 (0.39–10.36)	0.533
	Breed (crossbreed)	Purebred	2.54 (0.669–2.32)	0.242

## Data Availability

Not applicable.

## Supplementary Materials

The following supporting information can be downloaded at https://www.mdpi.com/article/10.3390/tropicalmed8040203/s1, Figure S1: Scatterplot depicting the relationship between seroprevalence tested via ELISA and VNT and average rainfall (mm/year) in the considered regions. A regression line for the two assays has also been superimposed.
