# Supplementary material for: Low Seroprevalence of WNV in Namibian Dogs Suggests a Limited Effectiveness as Sentinels for Infection Monitoring
Source: Trop Med Infect Dis. 2023 Mar 29;8(4):203. doi: 10.3390/tropicalmed8040203 (PMC10146826; doi:10.3390/tropicalmed8040203)
Supplement: Supplementary file 1 [file tropicalmed-08-00203-s001.zip › tropicalmed-2255968-supplementary.pdf]

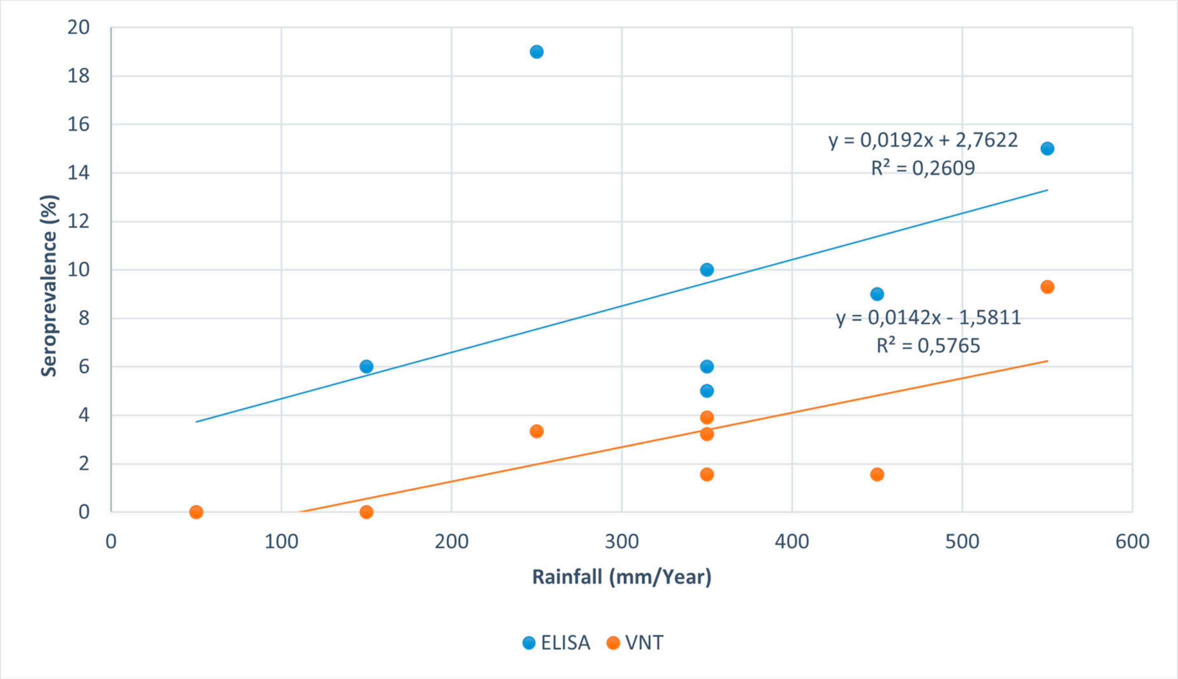


**Figure S1.** Scatterplot depicting the relationship between seroprevalence tested via ELISA and VNT and average rainfall (mm/year) in the considered regions. A regression line for the two assays has also been superimposed.
